# Development and Implementation of the AIDA International Registry for Patients With Still's Disease

**DOI:** 10.3389/fmed.2022.878797

**Published:** 2022-04-07

**Authors:** Antonio Vitale, Francesca Della Casa, Giuseppe Lopalco, Rosa Maria Pereira, Piero Ruscitti, Roberto Giacomelli, Gaafar Ragab, Francesco La Torre, Elena Bartoloni, Emanuela Del Giudice, Claudia Lomater, Giacomo Emmi, Marcello Govoni, Maria Cristina Maggio, Armin Maier, Joanna Makowska, Benson Ogunjimi, Petros P. Sfikakis, Paolo Sfriso, Carla Gaggiano, Florenzo Iannone, Marília A. Dagostin, Ilenia Di Cola, Luca Navarini, Ayman Abdelmonem Ahmed Mahmoud, Fabio Cardinale, Ilenia Riccucci, Maria Pia Paroli, Elena Maria Marucco, Irene Mattioli, Jurgen Sota, Anna Abbruzzese, Isabele P. B. Antonelli, Paola Cipriani, Abdurrahman Tufan, Claudia Fabiani, Mustafa Mahmoud Ramadan, Marco Cattalini, Riza Can Kardas, Gian Domenico Sebastiani, Henrique A. Mayrink Giardini, José Hernández-Rodríguez, Violetta Mastrorilli, Ewa Więsik-Szewczyk, Micol Frassi, Valeria Caggiano, Salvatore Telesca, Heitor F. Giordano, Emmanuele Guadalupi, Teresa Giani, Alessandra Renieri, Sergio Colella, Giulia Cataldi, Martina Gentile, Alessandra Fabbiani, Ibrahim A. Al-Maghlouth, Bruno Frediani, Alberto Balistreri, Donato Rigante, Luca Cantarini

**Affiliations:** ^1^Department of Medical Sciences, Surgery and Neurosciences, Research Center of Systemic Autoinflammatory Diseases and Behçet's Disease Clinic, University of Siena, Siena, Italy; ^2^Department of Translational Medical Sciences, Section of Clinical Immunology, University of Naples Federico II, Naples, Italy; ^3^Rheumatology Unit, Department of Emergency and Organ Transplantation, University of Bari, Bari, Italy; ^4^Rheumatology Division, Faculdade de Medicina, Hospital das Clínicas, Universidade de São Paulo, São Paulo, Brazil; ^5^Rheumatology Unit, Department of Biotechnological and Applied Clinical Sciences, University of L'Aquila, L'Aquila, Italy; ^6^Rheumatology, Immunology and Clinical Medicine Unit, Department of Medicine, Università Campus Bio-Medico di Roma, Rome, Italy; ^7^Internal Medicine Department, Rheumatology and Clinical Immunology Unit, Faculty of Medicine, Cairo University, Giza, Egypt; ^8^Internal Medicine Department, Clinical Immunology and Rheumatology Unit, Newgiza University (NGU), Giza, Egypt; ^9^Department of Pediatrics, Pediatric Rheumatology Center, Giovanni XXIII Pediatric Hospital, University of Bari, Bari, Italy; ^10^Rheumatology Unit, Department of Medicine and Surgery, University of Perugia, Perugia, Italy; ^11^Department of Maternal Infantile and Urological Sciences, Sapienza University of Rome, Rome, Italy; ^12^Azienda Ospedaliera (AO) Mauriziano, Universita degli Studi di Torino, Academic Rheumatology Centre, Turin, Italy; ^13^Department of Experimental and Clinical Medicine, University of Florence, Florence, Italy; ^14^Rheumatology Unit, Azienda Ospedaliero-Universitaria S. Anna - Ferrara, Department of Medical Sciences, University of Ferrara, Ferrara, Italy; ^15^University Department PROMISE “G. D'Alessandro”, University of Palermo, Palermo, Italy; ^16^Rheumatology Unit, Department of Medicine, Central Hospital of Bolzano, Bolzano, Italy; ^17^Department of Rheumatology, Medical University of Lodz, Łódź, Poland; ^18^Antwerp Unit for Data Analysis and Computation in Immunology and Sequencing, University of Antwerp, Antwerp, Belgium; ^19^Antwerp Center for Translational Immunology and Virology, Vaccine and Infectious Disease Institute, University of Antwerp, Antwerp, Belgium; ^20^Department of Paediatrics, Antwerp University Hospital, Antwerp, Belgium; ^21^Center for Health Economics Research and Modeling Infectious Diseases, Vaccine and Infectious Disease Institute, University of Antwerp, Antwerp, Belgium; ^22^Joint Academic Rheumatology Program, First Department of Propedeutic Internal Medicine, School of Medicine, National and Kapodistrian University of Athens, Athens, Greece; ^23^Rheumatology Unit, Department of Medicine, University of Padua, Padua, Italy; ^24^Uveitis Unit, Department of Sense Organs, Eye Clinic, Sapienza University of Rome, Rome, Italy; ^25^Division of Rheumatology, Department of Internal Medicine, Gazi University Faculty of Medicine, Ankara, Turkey; ^26^Ophthalmology Unit, Department of Medicine, Surgery and Neurosciences, University of Siena, Siena, Italy; ^27^Pediatric Clinic, University of Brescia and Spedali Civili di Brescia, Brescia, Italy; ^28^U.O.C. Reumatologia, Ospedale San Camillo-Forlanini, Rome, Italy; ^29^Vasculitis Research Unit and Autoinflammatory Diseases Clinical Unit, Department of Autoimmune Diseases, Hospital Clinic of Barcelona, Institut d'Investigacions Biomédiques August Pi i Sunyer (IDIBAPS), University of Barcelona, Barcelona, Spain; ^30^Department of Internal Medicine, Pulmonology, Allergy and Clinical Immunology, Central Clinical Hospital of the Ministry of National Defence, Military Institute of Medicine, Warsaw, Poland; ^31^Rheumatology and Clinical Immunology, Spedali Civili, Department of Clinical and Experimental Sciences, University of Brescia, Brescia, Italy; ^32^Azienda Socio-Sanitaria Territoriale (ASST) Gaetano Pini-Centro Traumatologico Ortopedico (CTO), Department of Clinical Sciences and Community Health, Research Center for Adult and Pediatric Rheumatic Diseases, University of Milan, Milan, Italy; ^33^Medical Genetics, Department of Medical Biotechnologies, University of Siena, Siena, Italy; ^34^Department of Medical Biotechnologies, Med Biotech Hub and Competence Center, University of Siena, Siena, Italy; ^35^Genetica Medica, Azienda Ospedaliero-Universitaria Senese, Siena, Italy; ^36^Department of Family and Community Medicine, College of Medicine, King Saud University, Riyadh, Saudi Arabia; ^37^Unit of Rheumatology, Azienda Ospedaliero-Universitaria Senese, Siena, Italy; ^38^Bioengineering and Biomedical Data Science Lab, Department of Medical Biotechnologies, University of Siena, Siena, Italy; ^39^Department of Life Sciences and Public Health, Fondazione Policlinico Universitario A. Gemelli IRCCS, Rome, Italy; ^40^Rare Diseases and Periodic Fevers Research Centre, Università Cattolica Sacro Cuore, Rome, Italy

**Keywords:** autoinflammatory diseases, precision medicine, personalized medicine, rare diseases, research, treatment

## Abstract

**Objective:**

Aim of this paper is to present the design, construction, and modalities of dissemination of the AutoInflammatory Disease Alliance (AIDA) International Registry for patients with systemic juvenile idiopathic arthritis (sJIA) and adult-onset Still's disease (AOSD), which are the pediatric and adult forms of the same autoinflammatory disorder.

**Methods:**

This Registry is a clinical, physician-driven, population- and electronic-based instrument implemented for the retrospective and prospective collection of real-world data. The collection of data is based on the Research Electronic Data Capture (REDCap) tool and is intended to obtain evidence drawn from routine patients' management. The collection of standardized data is thought to bring knowledge about real-life clinical research and potentially communicate with other existing and future Registries dedicated to Still's disease. Moreover, it has been conceived to be flexible enough to easily change according to future scientific acquisitions.

**Results:**

Starting from June 30th to February 7th, 2022, 110 Centers from 23 Countries in 4 continents have been involved. Fifty-four of these have already obtained the approval from their local Ethics Committees. Currently, the platform counts 290 users (111 Principal Investigators, 175 Site Investigators, 2 Lead Investigators, and 2 data managers). The Registry collects baseline and follow-up data using 4449 fields organized into 14 instruments, including patient's demographics, history, clinical manifestations and symptoms, trigger/risk factors, therapies and healthcare access.

**Conclusions:**

This international Registry for patients with Still's disease will allow a robust clinical research through collection of standardized data, international consultation, dissemination of knowledge, and implementation of observational studies based on wide cohorts of patients followed-up for very long periods. Solid evidence drawn from “real-life” data represents the ultimate goal of this Registry, which has been implemented to significantly improve the overall management of patients with Still's disease. NCT 05200715 available at https://clinicaltrials.gov/.

## Introduction

Data available on rare disorders are mainly excerpted from case reports, case series and small observational studies. This represents a harsh struggle for physicians and researchers dedicated to such diseases, as recruiting a sufficient number of patients may be challenging. A direct consequence is the lack of solid evidence on long-term disease course, borderline or atypical clinical manifestations, proper clinical management, short and long-term outcomes, prognostic factors, and the most appropriate therapeutic solutions. This is also evident for patients with autoinflammatory disorders (1, 2).

As a matter of fact, new research tools based on the Internet are going to overcome traditional research approaches in the field of rare diseases; patients' registries have taken a first-in-charge position among new electronic tools due to their capacity to recruit numerous patients followed-up for very long periods. The primary importance of patients' registries has also been recognized by the European Union, which has included these tools among the effective strategies to implement for rare diseases and has also provided guidelines aimed at ensuring high quality registries (3–5).

As a whole, these reasons have brought about the development of an international platform hosting specific registries dedicated to monogenic and multifactorial autoinflammatory diseases. This project has taken the name of AIDA from the acronym of AutoInflammatory Disease Alliance. The primary objective of the project has been the creation of an International Network of researchers and physicians interested in sharing knowledge and expanding current evidence about autoinflammatory diseases, including systemic juvenile idiopathic arthritis (sJIA) and adult-onset Still's disease (AOSD), which are the pediatric and adult forms of the same autoinflammatory disorder. The AIDA Network may be reached at the following website: https://aidanetwork.org/en/.

AOSD and sJIA are rare diseases characterized by the triad of daily spiking fever, arthritis, and evanescent salmon-colored skin rash, but serositis, lymphoadenopathy, hepatomegaly, splenomegaly, and lung inflammatory involvement may also be encountered (6, 7). Life-threatening complications, such as macrophage activation syndrome (MAS) and interstitial lung disease, may complicate both diseases (8, 9).

Laboratory investigations typically show an elevated white blood cell count with neutrophil predominance, increased inflammatory markers, and high levels of serum ferritin. Serum liver enzymes are also increased in some patients (10). To date, diagnosis of AOSD and sJIA are clinical and require the exclusion of infectious, neoplastic and autoimmune diseases. Different sets of criteria have been developed for diagnostic and classification purposes, with Yamaguchi's criteria and Fautrel's criteria being the most frequently employed for adult patients (11, 12), while the Pediatric Rheumatology INternational Trials Organization (PRINTO) provisional criteria and the International League of Associations for Rheumatology (ILAR) criteria are used for pediatric patients (13).

In this paper we are going to illustrate the steps that led to the development and activation of the International AIDA Registry conceived for patients with Still's disease, focusing on the rationale, design, material, and methods employed along with the diffusion of the project.

## Materials and Methods

### Study Design

This AIDA Registry has been thought as an international, clinical, physician-driven, population- and electronic-based registry for patients diagnosed with Still's disease, disregarding the age at disease onset.

Data collection includes both a retrospective and a prospective phase. The former refers to demographic, clinical laboratory and therapeutic data accrued up to the time of enrollment in the Registry; the latter is about progressive updates in clinical, therapeutic, and socioeconomic conditions reported thereafter. The prospective data collection consists of regular updates (at least one per year), but is particularly recommended when changes in treatment options, including dosage modifications and different molecules combinations, occur.

As part of its observational design, the Registry requires the collection of demographic, genetic, clinical, laboratory and treatment data collected over the past months/years of disease activity and over the future years of disease. Data will be exclusively captured from the routine assessments performed in the context of the standard management included in the daily clinical practice and no additional information will be requested. Furthermore, all of the therapeutic choices and eventual treatment changes proposed to patients will not be affected by adherence to the project itself, but will only be guided by physicians' clinical judgment to preserve and improve patients' health.

Participation in the AIDA project is free and open to any Center that deals with the management, diagnosis, and treatment of pediatric or adult-onset Still's disease; no limits as to the clinical specialty, location, and type of practice setting have been provided and no costs or financial fees are settled, since data inserted are usually collected throughout standard practice. As a prerequisite for adherence to the project, each Center should obtain approval from the local Ethics Committee and should define a Principal Investigator and at least a Site Investigator, which will, respectively, manage the local coordination of the study and documentation at data entry. After having presented a formal request about the involvement in the AIDA Network to the study Promoter, all Centers receive the proper credentials to access the Registry and start patients' enrollment.

### Registry Objectives

The Registry for Still's disease is primarily intended to gather as much data as possible from a robust cohort of patients enrolled on an international basis, in order to homogenize the research efforts and obtain significant results from real-world experience, disregarding specific geographic contexts. The first research paper obtained from data recruited in this Registry will focus on better characterizing prognostic factors capable of identifying patients more likely to develop complications in the short- and long-term. Future objectives would include matching the best treatment approach with patient's characteristics.

Other objectives of this Registry are: (a) to identify disease features in the light of a possible evolution toward different patterns of disease course based on the current diagnostic and therapeutic acquisitions; (b) to look for any change in the prognosis in relationship with an earlier diagnosis owing to a better knowledge and awareness of this disease; (c) to try to cluster disease features in order to identify subgroups of patients showing different prognosis or requiring different treatment strategies; (d) to highlight differences in the modalities of disease expression and severity according to the geographical context; (e) to identify any possible predisposing factors and triggers responsible for the onset and the acute exacerbation of the disease, quantifying and layering the intensity of the manifestations and response to treatments; (f) to describe old and new therapeutic regimens, specifically focusing on their global efficacy and their impact on different features of the disease; (g) to define a treat-to-target strategy in relationship with how to use corticosteroids, conventional immunosuppressants and biotechnologic agents in the earliest phase of disease; (h) to evaluate the best timing to start biotechnologic treatment in order to improve prognosis and induce a long-term remission; (i) to carefully study posologies and their adjustments to create standardized treatment protocols; (j) to look for evidence on the tapering and withdrawal of treatment strategies for any of the therapeutic approaches currently employed (especially conventional immunosuppressants, interleukin-1 and interleukin-6 inhibitors); (k) to assess the socioeconomic influence of the disease in terms of access to healthcare and patients' absenteeism due to the disease; (l) to identify different diagnostic strategies fitting with regional areas and evaluating the treatment response according to the resources available worldwide; (m) to better characterize the behavior of the disease during pregnancy and the trend of disease activity during the postpartum; (n) to monitor the cardiovascular risk in patients with Still's disease; (o) to identify clinical and biological factors predisposing to MAS development, which is the most frequent life-threatening complication of Still's disease; (p) to explore the therapeutic options and results about the pharmacological agents used in this severe condition; (q) to assess the reproducibility (sensitivity/specificity) of the different classification/diagnostic criteria currently used for sJIA and AOSD.

Finally, pioneering studies may eventually be designed according to the population extent of patients enrolled, with the perspective of selecting patients that may fit to future Randomized Control Trials (RCTs), whose realization is nowadays challenging because of the low epidemiological disease impact worldwide. Table 1 summarizes primary, additional and ancillary objectives of this Registry.

**Table 1:** List of the objectives that have driven the implementation of the AIDA Registry for patients with Still's disease.

Primary objectives		To gather as much data as possible from a large cohort of patients enrolled on an international basis
		To obtain real-world experience applicable to all geographic contexts
		To identify prognostic factors capable of tapering patients' management and treatment in the light of a personalized medicine approach
Additional objectives	Regarding diagnosis	To highlight disease differences in the severity and modalities of presentation of the disease according to the geographical context
		To assess the reproducibility (sensitivity/specificity) of the different classification/diagnostic criteria currently used for sJIA and AOSD
		To cluster disease features in order to identify subgroups of patients with different prognosis or requiring different treatment strategies
	Regarding prognosis	To look for any impact of diagnostic delay on disease prognosis
		To identify any possible predisposing factor and trigger inducing disease exacerbations
		To better characterize the behavior of the disease during pregnancy and postpartum period
		To monitor the cardiovascular risk, adjusting for treatments employed
		To identify clinical and biological factors predisposing to MAS development, which is the most frequent life-threatening complication of Still's disease
		To assess whether and how disease course has changed due to the current diagnostic and therapeutic evolution
	Regarding therapy	To define a treat-to-target strategy regarding how to use corticosteroids, conventional immunosuppressants and biotechnologic agents in the earliest phase of the disease
		To evaluate the best timing to start biotechnologic treatment
		To assess starting posologies and posologies adjustments
		To look for evidence on the tapering and withdrawal of treatment strategies
		To assess the socioeconomic impact of the disease before and after treatment
		To explore the therapeutic options and results about the pharmacological agents used in this severe condition
		To identify different diagnostic strategies fitting with regional areas and evaluating the treatment response according to the resources available worldwide
		To describe old and new therapeutic regimens, specifically focusing on their global efficacy and role on the different features of the disease
Ancillary objectives		To quickly find patients to be potentially included in randomized controlled trials
		To think about retrospective and prospective studies capable of answering future unmet needs

*Primary objectives have been distinguished from additional objectives: the former represents the general purposes at the basis of the Registry development, while the latter consist of the main lines of research the AIDA Network will follow in next years*.

### Inclusion/Exclusion Criteria

Patient's inclusion into the Registry strictly requires the fulfillment of Yamaguchi's criteria and/or Fautrel criteria and/or Cush criteria (11, 12, 14). Patients with pediatric disease onset (<16 years old) have to fulfill the International League of Associations for Rheumatology (ILAR) criteria for sJIA and/or the Pediatric Rheumatology INternational Trials Organization (PRINTO) provisional criteria for sJIA (13, 15).

Moreover, the patient has to provide her/his written and informed consent after a previous detailed explanation from the referring physician. The physician should carefully inform the patient about the project and its aims; the absence of implications of the study on her/his own clinical management; the free choice to deny the consent without this may affect the relationship with the reference Center; the international laws guaranteeing patients' privacy, anonymity and security of data, in line with the local and/or European legislation; and the chance to withdraw from the project at any time.

For minor patients or patients unable to provide their consent, this should be given by parents or legally authorized representatives, as long as they will observe the study requirements highlighted in the protocol for the entire duration of the study. No other exclusion criteria or conditions are previewed for the enrollment.

Patients not fulfilling diagnostic and classification criteria for AOSD and sJIA along with subjects who will not fully and freely agree with the project can not be recruited in the Registry dedicated to Still's disease.

### Online Data Collection

The Research Electronic Data Capture (REDCap) tool has been employed to collect and store data for the AIDA Project. REDCap is an electronic data collector produced at Vanderbilt University Medical Center (VUMC) and currently residing at the Virginia Commonwealth University (Award Number UL1TR002649). The access to the REDCap platform is free to all members of the REDCap consortium, which may use the tool in exchange for technical support. To date, over 5800 worldwide institutions from 145 countries already take part in the REDCap consortium (16). The access to the Registry website (at page: https://sitbio.med.unisi.it/redcap/redcap_v12.2.1/index.php?pid=41) is password-protected and the recruited information is stored on the servers of the University of Siena, Siena, Italy. The Registry may be reached via REDCap web interface using the private credentials supplied to each Principal and Site Investigator. The Registry's browser interface provided for data entry is entirely supplied in English in order to facilitate collection and reduce any language barriers. Privacy is granted for each Center's data: Principal and Site Investigators of a given Center cannot access the information collected by other Centers.

Variables included in the Registry depends on the fixed objectives. On this assumption, the number and nature of data elements included have been carefully determined based on the literature analysis and the evaluation of current unmet needs. The number of variables has been determined considering the costs of data collection, the potential burden of missing data, any loss of investigators' compliance, but also the need for a high detailed and specialized research and the will to develop an all-inclusive scientific tool.

In order to enhance registry feasibility and sustainability, variables included in the Registry have been distinguished into “mandatory” and “should have”, with the former being data to be collected compulsory and the latter being desirable, but not essential.

### Data Quality Management

Central to the development of a registry is maintenance of a high quality of the data entered, which is essential to obtain robust information and definitive study results (17). When developing this Registry, many precautions have been adopted to ensure data quality: quality assurance, quality control, and quality improvement. Quality assurance refers to the activities aimed at obtaining the highest quality of data that have preceded data collection, including the search for the essential variables required to describe patients with Still's disease and the critical revision of such variables. Quality assessment refers to periodical revisions of data included in the Registry, to minimize missing data and avoid discrepancies in data collected. Quality improvement consists of a constant effort to keep up to date the variables required to answer to address the future unmet needs. Also, Site Investigators will be continuously trained to collect and enter data in the most correct and complete manner possible.

### Ethics

In June 2019 the Ethics Committee of the Azienda Ospedaliero-Universitaria Senese, Siena, Italy (Ref. N. 14951; NCT05200715) granted the first national regulatory approval. After that, Centers experienced in diagnosis, clinical management and treatment of AOSD from Europe, the Middle East, the far East, Africa and North and South America have been invited to approve the project in order to join the AIDA Network.

Patients' data are kept in accordance with the EU General Data Protection Regulations (GDPR), or other counterparts, on the processing of personal data and the protection of privacy (2016/679/EU) (18).

The Registry protocol meets the recommendations from the Declaration of Helsinki. In particular, patients enrolled have to give their voluntary informed consent; otherwise, assent is required from minor patients aged ≥ 12 years or when the participant is not competent to provide the consent. In these last cases, parents/legal guardians have to give their approval to be part of the project.

Consent for the use of data for statistical analyses may be withdrawn at any time by patients or Principal Investigators. If the patient revokes the consent, no more data will be collected into the Registry; moreover, the patient has the right to obtain the erasure of personal data. In this regard, all data already gathered in the Registry will be deleted soon after the patient's notification to the study Promoter.

Participation in the study does not involve any kind of financial remuneration neither for the patient nor for the physician or Center, and there should be no evidence of any billing relationships with the national health system or insurance companies.

### Statistical Analysis

Statistical analysis will depend on the specific goals to pursue. However, the analysis will embrace general principles of descriptive statistics, correlations between groups and comparisons between subgroups. Also learning machine systems will be used in the future to enhance real-world evidence.

An unacceptable level of missing data is set to 25%. Variables not reaching at least 75% of compilation will be excluded from statistical analysis. For variables reaching a higher than 75% level of compilation, pair-wise deletion will be used to manage missing data, basing on the assumption that lacking data are completely missing at random (the probability that data are missing is not related to either the specific value which is supposed to be obtained or to the set of observed responses) or missing at random (the probability that the responses are missing depends on the set of observed responses, but is not related to the specific missing value which is expected to be obtained) (19).

## Results

The creation and activation of this AIDA Registry is a first fundamental result of the AIDA project. Actually, the development of this Registry fulfills the main purpose to create an online tool capable of gathering real-world data aimed at obtaining strong scientific evidence through the recruitment of a large number of patients diagnosed with Still's disease.

**Figure 1:**
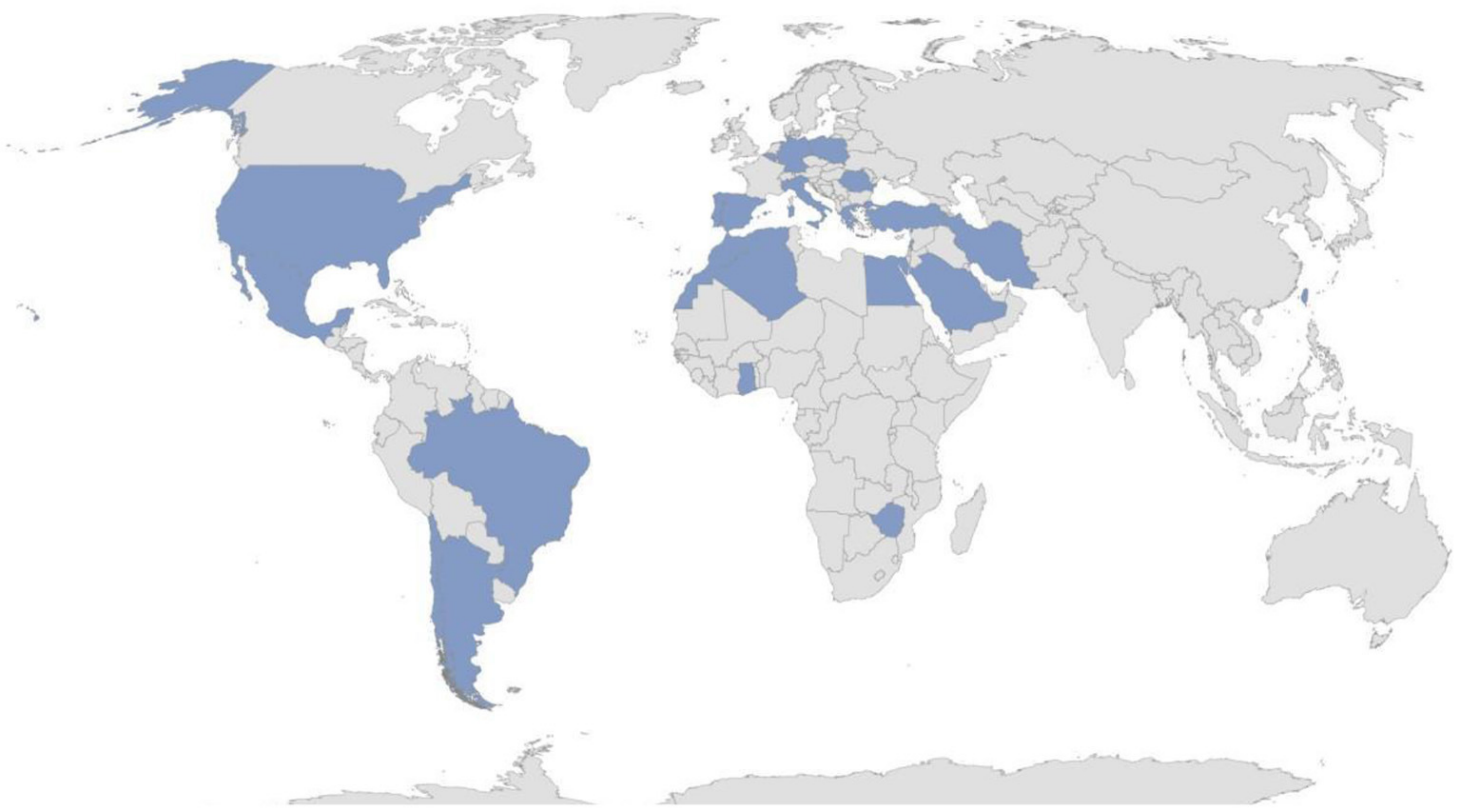
Worldwide distribution of the AIDA network in February 2022.

That being so, 23 nations distributed in 4 continents (Algeria, Argentina, Belgium, Brazil, Chile, Egypt, Germany, Ghana, Greece, Iran, Italy, Lebanon, Mexico, Morocco, Poland, Portugal, Romania, Saudi Arabia, Spain, Taiwan, Turkey, United States, Zimbabwe) have already joined the AIDA Network. Figure 1 highlights the worldwide distribution of the AIDA network. Overall, 110 Centers around the world have joined the project; 20 of those have currently (February 14th, 2022) entered data on the Registry; 290 users (111 Principal Investigators, 175 Site Investigators, 2 Lead Investigators, 2 Data Managers) have applied for credentials to access the Registry.

At present, 178 patients (74 males/104 females) with Still's disease have been enrolled in the Registry in about 8 months from the activation (June 30th, 2021).

### Registry Development

When establishing clinical variables to include in the Registry, it was pursued the ultimate purpose of comprehensively tracing the whole clinical and therapeutic history of the patient enrolled, in order to provide answers to the unmet needs deriving from current clinical practice. To date (February 14th, 2022), the Registry consists of 4,449 common data elements (each representing a study variable) organized into 14 instruments. While 9 instruments are dedicated to the retrospective phase, 4 instruments are built for both retrospective and prospective phases; the last instrument is specifically intended for longitudinal data collection. The Instruments included in the Registry and the corresponding phases (i.e., retrospective/prospective) at which they should be referred to are shown in Table 2.

**Table 2:** List of instruments included in the Registry dedicated to patients with Still's disease, with the corresponding number of common data elements, the phase (i.e., retrospective/prospective) at which they should refer to and the number of mandatory variables included.

**Instruments**	**Variables**	**Retrospective/prospective phase**	**N. of mandatory variables**
Demographics	11	Retrospective phase	4
Consents	4	Retrospective phase	1
Diagnostic data and family history	25	Retrospective phase	2
Clinical and laboratory features of Still's disease	160	Retrospective phase	0
Clinical diagnostic scores and criteria	16	Retrospective phase	1
Cardiovascular risk	24	Retrospective/prospective phase	2
Past and current treatments	1	Retrospective/prospective phase	0
Corticosteroids as monotherapy/main therapy—the retrospective phase	227	Retrospective phase	1
Treatment with cDMARDs not associated to biologic agents—the retrospective phase	591	Retrospective phase	6
Treatment with small molecules not associated to biologic agents—the retrospective phase	1048	Retrospective phase	12
Treatment with biotechnological agents—the retrospective phase	1212	Retrospective phase	14
Fertility and pregnancy	14	Retrospective/prospective phase	1
Disease course and treatment during pregnancies	66	Retrospective/prospective phase	1
Follow-up visits: clinical manifestations and treatment—the prospective phase	897	Prospective phase	51

### Registry Structure and Organization

Common data elements consist of demographic, instrumental, laboratory, therapeutic and any other clinical variable useful to completely describe patients' history. In particular, variables are organized to define family history, symptoms and clinical/laboratory signs at disease onset, symptoms developed during patient's history, Still's disease classification criteria (11–15), genetic features (including human leukocyte antigens and genes not related to the most common autoinflammatory diseases, such as CSF1 and IL18, suggested to be associated to Still's disease), comorbidities, cardiovascular risk, detailed information about treatments, including dosage changes, combinations, withdrawals or additions carried out over time. Data about disease course during and soon after pregnancy, long-term clinical outcomes and access to health care have also been included. Both the retrospective and prospective instruments require laboratory parameters such as daily routine investigation and more specific laboratory exams (lactate dehydrogenase, β2-microglobulin, ferritin serum level, percentage of glycosylated ferritin and 24 h-proteinuria). The filling-in of the following clinimetric scores: Pouchot score and modified Pouchot score by Rau et al. (10, 20), visual analog scale (VAS) for articular pain, patient global assessment (PGA), evaluator's global assessment (EGA), Health Assessment Questionnaire score (HAQ) or childhood HAQ (CHAQ); disease activity score based on 28 joints (DAS28) calculated with erythrocyte sedimentation rate (ESR) and with C reactive protein (DAS28-CRP) or juvenile disease activity score on 27 joints (JADAS27) with ERS and CRP. Laboratory parameters include daily routine investigation, such as inflammatory markers, liver enzymes and 24 h proteinuria. In addition, more specific laboratory exams are required: lactate dehydrogenase, β2-microglobulin, ferritin serum level and percentage of glycosylated ferritin.

Using a branching mechanism, the various fields are organized in such a way as to appear only when clinical history makes it necessary. In this way, only a few parts of the 4310 fields will appear during data entry, and the number of questions the investigator will have to answer is closely related to the complexity of patient's clinical history.

Many common data elements are shared with other AIDA registries dedicated to different autoinflammatory diseases, enhancing the merging of data among different Registries and the consequent optimal use of information for different research projects.

### Patients' Involvement

During the last decades patients have become aware of the importance in stimulating and supporting research. Patients have an active and pivotal role also in this project, as they may advocate the participation of Centers, help and support recruitment providing their own time, enhance data recruitment supplying patients reported outcomes as well as past information, and support a further diffusion of the project. For these reasons, patients' associations can be of outstanding help, as happens for ANMAR (Associazione Nazionale Malati Reumatici) and APMARR (Associazione Nazionale Persone con Malattie Reumatologiche e Rare), that are Italian associations of patients suffering from rheumatologic diseases.

Noteworthy, based on patients' suggestions, an electronic system for collecting patient-reported data (AIDA for patients) is under development. Among other things, AIDA for patients will also lead to a better data collection, minimizing the amount of missing values and substantially reducing the work burden for the Site Investigators, along with the risk for selection bias, the loss of prospective follow-up data and challenges resulting from physicians' time constraints.

## Discussion

Still's disease is a rare multifactorial autoinflammatory disorder mainly characterized by fever, skin manifestations (salmon-colored evanescent rash and/or heterogeneous atypical cutaneous lesions), arthralgia, arthritis, lymphadenopathy, liver involvement, serositis, neutrophilic leukocytosis and prominent increase of laboratory inflammatory markers and ferritin serum levels (15). Despite the good overall prognosis of the disease, life-threatening complications may sometimes occur, especially when macrophage activation syndrome (MAS) develops (8).

Diagnosis is based on the fulfillment of internationally accepted criteria to apply only after the exclusion of neoplastic, infectious, autoimmune, and other monogenic and multifactorial autoinflammatory diseases (11–15). AOSD is a very uncommon disease with an annual incidence estimated between 0.1 and 0.4 cases per 100,000 people in Europe (21). Also sJIA, which is considered the pediatric counterpart of AOSD, is a rare condition and may be encountered in about 10–20% of all cases of juvenile idiopathic arthritis (22).

As for other rare diseases, the low epidemiological burden of Still's disease determines major difficulties in scientific research, due to the limited number of patients available for RCTs or even for retrospective “real-life” studies. Therefore, gathering patients together through the new web-based technologies is an invaluable opportunity to perform cutting edge and ambitious studies capable of obtaining solid results, also in the field of Still's disease. Noteworthy, this Registry is not only intended to enable a broad population-based data collection, but also to stimulate the scientific community in focusing research efforts on specific targets reflecting the current unmet needs in the clinical practice. The project includes patients disregarding the age at disease onset and age at the enrollment.

The Registry represents a potential opportunity to assess the performance of currently available classification criteria in different geographic realities and to eventually elaborate new diagnostic/classification criteria for sJIA and AOSD specifically tailored on patient subsets or contexts. Looking at the clinical management of patients with Still's disease, many doubts about proper care and treatment should be solved at present. For instance, the Registry would provide valuable information about how to taper the different treatment strategies in patients with Still's disease. In this regard, the identification of predictive variables capable of correlating with disease relapses after drug tapering or withdrawal is crucial to establish whether and when to successfully reduce treatments.

The increasing number of therapeutic opportunities for patients with Still's disease has paved the way to the possible identification of treatment protocols tailored on genetic, laboratory and clinical patients' features. This could be part of a personalized medicine model specifically thought for patients suffering from Still's disease. Also, the identification of predictive variables capable of early detecting the different patterns of the disease, long-term outcomes, and any development of complications may further contribute to outline a personalized medicine approach for such condition.

Of note, only little information is available about the behavior of Still's disease during pregnancy or breastfeeding. Despite the significant efforts to better characterize Still's disease during these periods, only a few data are available regarding: (a) the timing of disease flares during pregnancy and postpartum period; (b) the different possible patterns of disease course (previously reported as first-onset type, recurrent-flare type, no-flare type) (23); (c) the best diagnostic and therapeutic approaches for first-onset disease and new flares in patients with polycyclic course; (d) the major complications possibly affecting the pregnant, the fetus and the newborn; (e) the comprehensive management of Still's disease during pregnancy and post-partum period.

The international patients' recruitment will allow the assessment of any possible change in the clinical behavior, course, prognosis and treatment response in the light of the latest treatment acquisitions and according to the specific geographical and ethnic contexts. Moreover, the sensibility and specificity of the internationally accepted classification criteria for Still's disease will be assessed according to the different settings (11–15).

Currently existing registries for patients with Still's disease are almost nation-based or borrowed from other registries created to collect information about biologic treatments. Among the others, the following projects account for some of the available registries, especially designed for pediatric patients: the UK juvenile idiopathic arthritis biologic registry, pharmacovigilance in juvenile idiopathic arthritis, the Turkish Pediatric Rheumatology Association registry, the Childhood Arthritis and Rheumatology Research Alliance (CARRA) registry, the German biologic registers including the German biologics in pediatric rheumatology (BIKER) registry, the JuMBO (Juvenile Arthritis MTX/Biologics Long-Term Observation) registry, the autoinflammatory disease (AID) registry as part of the Network for autoinflammatory diseases funded by the German Federal Ministry of Education and Research (24–28). Despite these existing registries, the AIDA Registry for Still's disease is aimed at collecting data from many clinical and research perspectives with no age limitations and with the ambitious purpose to carefully report clinical history of the patients enrolled. The Registry is also intended to improve the routine patients' management, as some of the variables included are thought to investigate the best standard of care according to patients' features. The instrument dedicated to prospective follow-up visits could be also used in the clinical setting during routine visits not only to collect prospective data, but also as a guide for clinical management. In this regard, the compilation of the follow-up instrument requires from 5 to 10 min, which may perfectly fit with the visiting time.

The international basis of data recruiting is also aimed at overcoming the geographical differences due to ethnicities, environmental features and specific health strategies. In this way, it will be possible to generalize the results thanks to the wide sample size.

In thinking about this Registry, recommendations and practical guidelines provided to consider the methodological and operational aspects of patient registries were carefully followed (4, 29, 30). These practical guidances designed to consider all aspects of planning and executing patient registries helped overcome many of the obstacles and pitfalls associated with the development of this Registry.

The AIDA Registry for patients with Still's disease shows the typical limits of observational studies regarding the completeness and accuracy of data collection. At the same time, the investigators are not obliged to consecutively enroll all patients with Still's disease referred to their center; as a consequence, this may lead to unintended selection bias. Furthermore, this Registry will include only patients fulfilling currently available diagnostic/classification criteria. This may lead to the exclusion of patients with atypical Still disease. Nevertheless, three diagnostic criteria for adult patients and two classification criteria for pediatric patients have been considered for inclusion criteria in this Registry, thus minimizing the percentage of patients that will be excluded from the enrollment. Patients with suspected Still disease, but with no diagnostic/classification criteria fulfilled, should be included in the registry dedicated to USAIDs for future and specific analysis aimed at the development of new or revised classification criteria. Of note, entering data into the Registry requires time and attention, especially when the medical history is particularly complex and many treatments have been attempted over time. Physicians and patients have to be motivated to give their time for data collection; indeed, the accuracy of data recruitment in the retrospective phase of the Registry may require many hours and the direct presence and involvement of patients during data gathering. Nevertheless, beyond its limits, this Registry has the potential and geographical basis to really achieve all the purposes proposed. Moreover, the prospective phase of the project will guarantee the recruitment of complete and easy-to-obtain data for future studies.

## Conclusions

In conclusion, the International Registry for patients with Still's disease has been developed and activated for data sharing, international consultation, and knowledge diffusion. The main reasons for its deployment are to overcome the scientific and clinical fragmentation currently existing on this rare disease, and to perform solid and pioneering international studies based on wide cohorts of patients and real-world data. The final goal will be to obtain the best evidence capable of significantly improving the daily management of patients with Still's disease.

## Ethics Statement

The studies involving human participants were reviewed and approved by Azienda Ospedaliero-Universitaria Senese, Siena, Italy (Ref. No. 14951). Written informed consent to participate in this study was provided by the participants' legal guardian/next of kin.

## Author Contributions

AV wrote the first draft of the manuscript and conceived and designed the study and the Still's disease Registry. DR critically revised the manuscript. FD, GL, RP, PR, RG, GR, FL, EB, ED, CL, GE, MGo, MM, AM, JM, BO, PSfi, PSfr, CG, FI, MD, ID, LN, AAh, FC, IR, MP, EM, IM, JS, AAb, IA, and PC were involved in data recruitment in the Registry dedicated to patients with Still's disease. AT, CF, MR, MC, RK, GS, HGia, JH-R, VM, EW-S, MF, VC, ST, HGio, EG, TG, AR, SC, GC, MGe, AF, IA-M, and BF were included in the authorship as investigators from the top three contributor centers for any of the other AIDA Registries (excluding the Registry dedicated to VEXAS disease). AB is the bioengineer involved in the technical management of the platform and registries. LC conceived and designed the study and accounts for AIDA Registries Coordinator. Authorship has been established based on the number of data recruited in the AIDA Registries on February 7th, 2022. All authors contributed to the article and approved the submitted version.

## Conflict of Interest

The authors declare that the research was conducted in the absence of any commercial or financial relationships that could be construed as a potential conflict of interest.

## Publisher's Note

All claims expressed in this article are solely those of the authors and do not necessarily represent those of their affiliated organizations, or those of the publisher, the editors and the reviewers. Any product that may be evaluated in this article, or claim that may be made by its manufacturer, is not guaranteed or endorsed by the publisher.
